# How Clinicians Prenatally Discuss Management Options and Outcomes for Congenital Heart Disease

**DOI:** 10.1016/j.jpainsymman.2025.11.026

**Published:** 2025-11-27

**Authors:** Samantha Syme, Kelsey Schweiberger, Judy C. Chang, Ann Kavanaugh-McHugh, Nadine A. Kasparian, Robert M. Arnold, Kelly W. Harris

**Affiliations:** University of Pittsburgh School of Medicine, Pittsburgh, Pennsylvania, USA; Department of Pediatrics, University of Pittsburgh School of Medicine, Pittsburgh, Pennsylvania, USA; Department of Obstetrics, Gynecology, and Reproductive Sciences, University of Pittsburgh School of Medicine, Pittsburgh, Pennsylvania, USA; Department of Pediatrics, Vanderbilt University Medical Center, Nashville, Tennessee, USA; Heart and Mind Wellbeing Center, Heart Institute and Division of Behavioral Medicine and Clinical Psychology, Cincinnati Children’s Hospital Medical Center and Department of Pediatrics, University of Cincinnati College of Medicine, Cincinnati, Ohio, USA; Department of Geriatrics and Palliative Medicine, Icahn School of Medicine at Mount Sinai, New York, New York, USA; Department of Pediatrics, University of Pittsburgh School of Medicine, Pittsburgh, Pennsylvania, USA

**Keywords:** Palliative care, quality of life, fetal cardiology, congenital heart disease, prenatal diagnosis, mortality

## Abstract

**Context.:**

A prenatal diagnosis of complex congenital heart disease (cCHD) introduces significant emotional, social, and financial stress for families. Uncertainty about the future is a central source of distress for parents, particularly surrounding management options and anticipated outcomes. Little is known about how fetal cardiologists present management options, address anticipated outcomes, and offer supportive services.

**Objective.:**

To examine how fetal cardiology clinicians discuss management options, anticipated outcomes, and supportive services with parents following a prenatal diagnosis of complex congenital heart disease (cCHD).

**Methods.:**

Initial fetal cardiology consultations were audio-recorded, transcribed, and analyzed qualitatively. Deductive coding focused on management options (e.g., invasive intervention, comfort care), anticipated outcomes (e.g., quality of life, mortality risk), and supportive services (e.g., social work, peer support, palliative care).

**Results.:**

Five fetal cardiology clinicians from one tertiary care institution participated in 19 initial consultations. Management options discussed included invasive intervention and comfort care. Clinicians frequently described anticipated outcomes involving quality of life and mortality risk but rarely used those terms specifically. Families were most engaged during quality-of-life discussions. Palliative care as a consulting service was selectively introduced, often when the CHD diagnosis carried a higher risk of mortality. The service was frequently described as additional support.

**Conclusion.:**

Fetal cardiology consultations offer an important opportunity to support families navigating uncertainty following a prenatal diagnosis of CHD. Clinicians approached these conversations with empathy and a focus on long-term outcomes, though discussions about management options varied. There is an opportunity for increased presentation and integration of palliative care consultants as a longitudinal, family-centered resource, regardless of mortality risk, which may enhance supports available to families during this highly emotional period. Ongoing efforts to improve family-centered counseling and resources, such as access to perinatal palliative care, may help ensure families receive comprehensive, values-aligned information across the spectrum of care pathways.

## Introduction

The most common congenital anomaly is congenital heart disease (CHD), occurring in nearly 1% of neonates.^1^ Complex CHD (cCHD) occurs in about 25% of CHD cases and often causes life-threatening symptoms requiring invasive intervention.^2^ cCHD diagnoses are increasingly made prenatally;^3^ however, due to technological limitations, ongoing cardiac development, and physiology changes, the diagnosis and its clinical implications, including management strategy, can only be fully understood after birth.^4,5^

For expectant parents, prenatally diagnosed CHD elicits severe psychological distress and substantial social, emotional, financial, and other stressors.^6–9^ Uncertainty surrounding their child’s future is a pervasive concern.^10^ Given the uncertainty surrounding a prenatal diagnosis of cCHD and its psychological consequences for parents, optimizing communication about uncertainties, including discussion of various clinical options, is necessary. In this study, we aimed to understand how fetal cardiologists discuss management options, anticipated outcomes, and supportive services with expectant parents.

## Methods

This study is a secondary analysis of qualitative data from the initial consultation portion of a larger study, which investigated the lived experiences of parents from the time of prenatal diagnosis of CHD through the postpartum period.^11,12^ Prior analyses utilized the same transcripts from initial fetal cardiology consultations but focused on different aspects of these conversations, including dialogue about uncertainty and discourse styles.^11,12^ The current analysis focuses on discussions about management options, anticipated outcomes, and supportive services.

Participants were recruited from one tertiary care institution between May and August 2019. All clinicians who conducted initial fetal cardiology consultations were invited to participate. Pregnant individuals referred for an initial fetal cardiology consultation for suspected cCHD were approached immediately before the consultation. Individuals who indicated English was not their native language were eligible if an in-person interpreter was used or if the visit occurred in English. All participating individuals, including clinicians, pregnant individuals, and accompanying individuals, provided written and verbal consent prior to participation. Demographic data were collected prior to the consultation. This study was approved by the Vanderbilt University and University of Pittsburgh Institutional Review Boards (protocols 190532 and 21080073).

Fetal cardiology consultations were audio-recorded using a digital voice recorder and transcribed verbatim with identifying information removed. When an interpreter was used, the interpreter’s words in English were transcribed verbatim.

A secondary data analysis was conducted focusing on deductively created codes related to: 1) management options including invasive interventions for life-prolongation, comfort (hospice-directed) care, and pregnancy termination; 2) anticipated outcomes or prognosis, including quality of life and mortality risk; and 3) supportive services, including palliative care consults, social work, and peer support (Table 1). In this study, comfort care refers to care initiated after the birth of a neonate with a life-limiting condition to provide comfort in lieu of life-prolonging surgical or medical intervention.^13^ It is often used in lieu of the term hospice when discussing neonates. Due to a shortage of providers, prenatal palliative care consultations were limited by the study institution to fetuses diagnosed with single ventricle CHD or with expected prenatal or postnatal death from a cardiac lesion.

Two coders examined each transcript independently; any coding discrepancies were discussed and resolved with the mentorship of an expert qualitative researcher on the study team. *NVivo14* software (QSR International; Burlington, Massachusetts) was used to organize coding for analysis.

## Results

### Participant Characteristics

Table 2 presents participant characteristics. Of the 31 pregnant individuals approached, 19 participated (five declined, seven were ineligible after fetal echocardiogram ruled out CHD). Median gestational age at time of consultation was 26 weeks. Most participants self-identified as White and non-Hispanic (74%) and reported English as their primary language (79%). Most (74%) were accompanied by another person in the consultation (partner, family member, or friend). In approximately half (47%) of consultations, a diagnosis associated with a higher mortality risk was made (score >5 on Allan (2004) scale).^14^

Of the seven clinicians approached, five participated (one declined, one had no eligible consultations during the study period). Clinicians trained at five different institutions and had a median of 10 years of clinical experience.

Results from the thematic analysis are presented with abbreviated quotations. Full quotations with participant identifiers are presented in Table 3.

### Management Options

Of the three categories of management options in our *a priori* categories, only invasive interventions and comfort care were discussed in our recorded visits.

#### Invasive interventions for life prolongation:

Invasive intervention options were discussed in every consultation, and the vast majority of each consultation was spent discussing these options. Clinicians spent most of the visit time talking about cardiac anatomy and invasive interventions to address the anatomy such as surgery, interventional catheterization, or transplantation. Invasive intervention options were discussed in detail; providers often drew pictures and/or provided families with handouts detailing the procedures. As one clinician explained, “*The first intervention she would need if we get to intervening is something called the Blalock-Taussig shunt, and that*’*s basically just something that does exactly what this ductus does*... *it comes off of one of the first branches of the aorta and then is attached directly to the pulmonary artery.*”

#### Comfort care:

Comfort care was discussed in five of the 19 (26%) consultations. When introducing the possibility of pursuing comfort care after birth, clinicians alluded to but did not explicitly use the term “comfort care” (or “hospice”). Instead, they focused on explaining the choice to pursue comfort care as part of a broader discussion about goals of care, often as a secondary choice to invasive interventions. For example, “*That [not pursuing surgery or transplant] actually is a choice some families make. They don*’*t make it as much anymore because our numbers are better. But that is there too.*” Another clinician stated, “*If we see how the surgery goes and the baby*’*s doing very poorly, at that point, you can say, “You know what? I think we need to stop intervening.” And that*’*s okay too.*”

### Anticipated Outcomes

#### Quality of life:

In 16 (84%) of the 19 consultations, clinicians introduced the topic of quality of life when discussing prognosis. In 10 consultations, functional outcomes, including participation in sports and physical activity were discussed; for example, “*We may have to limit some things like football, but that doesn*’*t mean he can*’*t do other sports competitively.*” In seven consultations, longer-term neurocognitive outcomes were discussed, such as learning disabilities, college attendance, and independent functioning as an adult. Clinicians frequently discussed the future parents might be imagining for their children; “*I anticipate he*’*s going to go to school. I anticipate he*’*s going to get married. And I think he*’*s going to do whatever he wants to do in life.*”

As reported previously, families only spoke, on average, 11% of the words in each consultation (range: 2%–23%).^11^ Family members did, however, engage in discussions related to quality of life in over half of the consultations. For example, one father asked, “*Is she happy?*”, to which the fetal cardiologist responded: “*She is, there is so much more to her than this. We have to make sure there aren*’*t any surprises there besides this.*”

#### Mortality risk:

Mortality risk was mentioned either explicitly, or implicitly through survival statistics or prognosis discussion, in 14 (74%) of the 19 consultations. Of the five remaining consultations in which risk of mortality was not discussed, four included a cardiac diagnosis of lower mortality risk and one family already had a living child with CHD. When risk of mortality was discussed, a variety of words were used to refer to death; for example, “*The chance of surviving the operation is over 90%.*” Clinicians used the following terms from most to least frequently: “survive” or “survival” (used in 79% of the 14 consultations where prognosis was mentioned), “death” or “dying” (36%), “mortality” (27%), “alive” or “living” (21%), “intrauterine fetal demise” (7%), and “life expectancy” (7%). Clinicians explicitly discussed the impact of mortality risk on management options; for example, “*If she has a significant abnormality with her chromosomes that we don*’*t think would be survivable in the long term, then we wouldn*’*t recommend that she goes through the suffering of surgery.*”

### Supportive Services

#### Social work:

Social work was offered as a service during six of the 19 (32%) consultations and was normalized by providers. When discussed, clinicians stated social work would meet with the family and help with everything from insurance changes to finding a place to stay. Social workers were discussed as an integral part of their ongoing care team. When discussing social work, one clinician explained, “*We have social workers who meet with you to make sure that we take care of all your insurance papers and they check to see if there*’*s anything else you need to do all of this.*”

#### Peer support:

Peer support was offered in various formats in 14 (74%) of the appointments. Support groups, both virtual and in-person, along with peer support websites, were brought up by clinicians, typically at the end of the visit. One clinician emphasized the importance of peer support, saying, “*When [learning what life will be like], the experts of this are not the doctors, but the parents who take care of kids like this.*” Clinicians also often provided a list of frequently asked questions and answers as starting point for families to think about what to ask at future appointments.

#### Palliative care:

Palliative care as a consultation service was introduced by providers in eight (42%) of the 19 appointments, specifically in 67% of the appointments in which there was a diagnosis with a higher risk of mortality. Palliative care was presented as a potential source of additional support, or as the team that could assist with end-of-life care if needed after birth, including if the family chose to pursue comfort care. When introducing the palliative care team as a source of support, clinicians suggested families could choose to engage with this service later. For example, “*They just help you process things and figure out what you want to do and what resources are available to us.*” Clinicians in these discussions normalized involving the palliative care team and distinguished palliative care team from comfort care. For example, “*Most people when they think of a palliative care team, it*’*s just for end-of-life stuff* ... *But that*’*s not what they do here* ... *They also support* ... *they see every child who gets a heart transplant.*”

## Discussion

This study explored how management options, anticipated outcomes, and supportive care services are discussed in initial fetal cardiology consultations. Clinicians primarily discussed invasive interventions with less discussion of comfort care. Across various management pathways, mortality was mentioned in most consultations. Clinicians also offered social work and peer support to families, and they introduced palliative care as a consultation service in almost half of consultations.

As CHD survival continues to improve, with a current one-year mortality rate around 25%,^2^ more children are undergoing invasive intervention, with the median age at first surgical intervention now 0.6 years.^15^ We show here that, as anticipated, clinicians discussed invasive intervention in every consultation and spent much of the time explaining possible procedures in great detail.

In our study, fetal cardiology clinicians also normalized comfort care as a path some families pursue when consistent with their goals of care. Interestingly, clinicians introduced the topic without specific use of the terms “comfort care” or “hospice.”^16,17^Additionally, they did not seem to assess individual goals of care; involvement of the palliative care team could help facilitate such conversations.^18,19^

Termination was not discussed in any of the consultations, potentially due to gestational age and local legal restrictions. There may also be assumptions that this discussion was the responsibility of the referring obstetrician and those who came to prenatal cardiology appointments were committed to continuing the pregnancy. When a prenatal CHD diagnosis occurs, families report three main factors in deciding whether to terminate the pregnancy - moral/religious beliefs, quality of life outcomes, and the chance of survival.^20^ Our study is limited, as we did not assess whether families had discussions regarding termination prior to the recorded visits. While quality of life and mortality were discussed in these consultations, we also do not know how these conversations may have influenced participants’ postvisit decision-making.

Regardless of the management options discussed, clinicians consistently discussed anticipated outcomes. In the prenatal period, previous research shows parents rank discussing quality of life relating to CHD as significantly more important than clinicians.^20^ In our study, most initial prenatal fetal cardiology consultations (84%) included discussions of quality of life, potentially indicating a prioritization of parental concerns early in the relationship. Our finding that families frequently engaged in these discussions, and otherwise spoke rarely, highlights the importance of the topic.

In most consultations, mortality risk was discussed, in accordance with previously reported parental and clinician preferences.^20^ Clinicians mostly framed mortality positively, in terms of survival; words such as “death” were used less frequently. Using a positive frame to present anticipated outcomes has been shown to affect treatment decisions in other healthcare settings, despite the underlying meaning being equivalent.^18,19^ Clinician intentions and the implications associated with word choices are not known in this setting.

Both peer support and social work were frequently offered to families in alignment with previously reported parental preferences for support.^21^ Research shows perceived social support is a crucial factor in resiliency and coping, making discussion of both peer support and social work imperative following a new diagnosis of CHD.^22^

Palliative care as a subspecialty was discussed in nearly half of the initial consultations, either as a resource for support in the future or for assistance if comfort care was pursued after birth. It was nearly always introduced when the cardiac diagnosis carried a higher risk of mortality. Given limitations in the number of palliative care providers available at the study institution, this consultation pattern was expected. Current guidelines recommend discussing palliative care at the time of prenatal diagnosis, regardless of mortality risk;^16^ however, as many medical centers may have few or no pediatric palliative care providers available to prenatal patients, this option may not be possible for many. We highlight here the discrepancy between the guidelines and the dearth of palliative care providers to meet these guidelines.

In this study, when palliative care was introduced as a source of support for families, clinicians normalized involving the team, emphasizing this as a service offered to most families. They also at times explicitly stated the palliative care team would provide support for more than just end-of-life care. Surveying pediatric cardiologists, Balkin et al.^23^ found the most common barrier to referring families to palliative care was the fear of harming parents’ sense of hope. Our findings suggest fetal cardiologists may attempt to assuage such fears by normalizing palliative care’s involvement.^16,17^ Family reactions to this approach to introducing palliative care should be studied.

While this study notably included clinicians and families from only one institution, it is the first, to our knowledge, to look directly at fetal cardiology clinicians’ discussion of care pathways, including management options, anticipated outcomes, and supportive services. Many prior studies of fetal cardiology counseling have relied on surveys and qualitative interviews on reported practices, while our study analyzed audio-recordings of conversations between fetal cardiology clinicians and parents. Families receiving diagnoses associated with a range of mortality risks were included. Clinicians received training from a variety of institutions; however, with only five clinician participants, findings may not be reflective of wider practices. Generalizability may also be limited by the demographics of participating families. The ability to consult palliative care was also limited by institution guidelines.

In conclusion, we found that clinicians discuss options for invasive intervention at length during initial fetal cardiology consultations with some mention of comfort care as well. Across these management pathways, quality of life and mortality are often discussed, and families are offered social supports, including palliative care within the confines of provider availability. Given the uncertainties surrounding a prenatal diagnosis of CHD and the need for ongoing discussions about quality of life and outcomes, the integration of palliative care early after diagnosis may help with long-term support and psychological outcomes for families. Our exploration of this topic was limited by institutional availability of palliative care providers, highlighting the need for more training and recruitment in the field. While the themes identified here relate to CHD, they may also be applicable and important to perinatal palliative care more broadly.

## Figures and Tables

**Table 1:** Coding Framework

Topic	Code	Description

Management	Life-prolonging intervention	Description of medical or surgical intervention aiming to prolong life of the patient
	Comfort care	Explicit or implicit reference to comfort-focused end-of-life care after birth
	Termination	Explicit or implicit reference to pregnancy termination
	Choices	Clinician explicitly mentions a choice or decision that the parents can make in terms of management options
Prognosis	Quality of life	Description of anticipated quality of life and functional outcomes, including specific examples of how others with similar diagnoses are doing later in life
	Mortality	Explicit or implicit reference to survival, mortality risk, or death (in utero fetal demise or death of a neonate)
Support	Parent support	Discussion of peer support online or in person via written resources or real-time conversations
	Other subspecialties	Family support from other subspecialties potentially on the prenatal team, including palliative care
	Other resources	Support from other human resources, such as social work or general pediatrics

**Table 2:** Demographics

	N	%

Pregnant individuals (N = 19)		
English as primary language	15	79
Higher mortality risk diagnosis^a^	9	47
Gestation – median (IQR), weeks	26 (25–32)	
Race/Ethnicity:		
White (European)	14	74
White (Middle Eastern)	2	11
Black/African American	2	11
Hispanic/Latina	1	5
Fetal cardiology clinicians (*N* = 5)		
Time in field – median (IQR), years	10 (9–11)	

a Lower risk of mortality defined as score of ≤5; higher risk of mortality defined as score of >5 on the suggested CHD prognosis scale from Allan and Huggon.^14^

**Table 3:** Exemplary Quotations

Theme	Quotations

*Management options: invasive interventions for life prolongation*	“The first intervention she would need if we get to intervening is something called the Blalock-Taussig shunt, and that’s basically just something that does exactly what this ductus does. . . it comes off of one of the first branches of the aorta and then is attached directly to the pulmonary artery. Because the medicine that we use to keep this ductus open has to be given in an ICU setting, we couldn’t ever send you home with it, so we need some way to provide pulmonary blood. . . The second stage occurs at three to four months of age and that’s called the Glenn Operation. . . The final stage is called the Fontan that usually happens at three to four years of age.”
	- Clinician 2, Family 10
	“Many times, this type of surgery can be done in the newborn period by one of our three pediatric cardiac surgeons. Their job day in and day out is to do surgeries to help repair differences at the heart. About one in a hundred children can have something different with their heart and this is just one of those things that happens. This isn’t anything you did or didn’t do, but if indeed your baby does have a narrowing, I’m not sure he does, but just to know this isn’t your fault if he does have a difference. It’s just one of those things that’s pretty common. Surgery for a coarctation of the aorta can often be done by going in between my rib cage in the back, rather than doing an open-heart surgery to remove the area that’s too small and then bring the other ends together to make it.”
	- Clinician 4, Family 15
*Management options: comfort care*	“There are some families who say, “Well, neither of these [surgery or transplant] sounds very good to me.” Without those, we would lose him. Okay. That actually is a choice some families make. They don’t make it as much anymore because our numbers are better. But that is there too.”
	- Clinician 1, Family 1
	“If at any point you say, “You know what? I don’t want to do a painful surgery on my baby.” We can definitely see then. . . Sometimes families decide to deliver the baby, but not do anything. And that would be okay too. . . Some families want us to try, let’s just see how the surgery goes. And if we see how the surgery goes and the baby’s doing very poorly, at that point, you can say, “You know what, I think we need to stop intervening.” And that’s okay too. When we have babies that are very high risk, it’s sort of- it’s important to have kind of a good conversation between the clinical team and the family about what’s important to the family.”
	- Clinician 2, Family 6
*Anticipated outcomes: quality of life*	“How are [the diagnoses including CHD and hydrocephalus] going to affect her? One way or another, in terms of her being able to have surgery and her survival. It’s like what you said, how’s it going to affect her?”
	- Family 16, Clinician 3
	“And as far as long-term with physical activity, with an excellent repair, we’d expect him to be able to do lots of normal physical activities. If there’s a need for a pacemaker, then that would limit being able to do some sports. Because his coronary arteries will then move from their position, we may have to limit some things like football. But that doesn’t mean he can’t do other sports competitively. That doesn’t mean he can’t run around and play with his friends, doing physical activity, you know?”
	- Clinician 3, Family 21
	“Do you know who Shaun White is, the snowboarder?... He has Tetralogy of Fallot. . . So, there you go. . . And they didn’t know, exactly, before he was born, but look at how well Shaun White, how athletic he is.”
	- Clinician 5, Family 11
	“It’s one that we’re fairly good at fixing. And again, one that we have really good long-term success with. I anticipate he’s going to go to school. I anticipate he’s going to get married. And I think he’s going to do whatever he wants to do in life. ”
	- Clinician 2, Family 2
	“And the real questions are what happens after that? Of course, there’s so many questions about her. Is she going to be an artist? Is she going be a dancer?”
	- Clinician 1, Family 3
*Anticipated outcomes: mortality risk*	“The chance of surviving the operation is over 90%. And the two-year survival for heart transplantation is well over 80%.”
	- Clinician 1, Family 1
	“If she has a significant abnormality with her chromosomes that we don’t think would be survivable in the long term, then we wouldn’t recommend that she goes through the suffering of surgery.”
	- Clinician 3, Family 16
	“If the electrical system is injured, then that means the top and bottom chambers don’t talk to one another the way they should. It would mean for a pacemaker. I think that’s the biggest kind of highest complication that can happen. The likelihood of that is less than 5%. So, it’s low that it would happen, but it’s the one that has the most significant long-term effect if it happens. Most kids who’ve had the VSD closure by surgery have a normal long-term life expectancy.”
	- Clinician 4, Family 4
*Supportive services: peer support*	“When [learning what life will be like], the experts of this are not the doctors, but the parents who take care of kids like this. And I have parents who’ve gone through this, who are happy to talk with other parents about what it’s like and share how their children are doing, which I think helps a lot of people. Parents also do this on the web and there are organizations to support parents.”
	- Clinician 1, Family 13
	“The last thing in here is, if you come, I shouldn’t say last, that same group, the children’s by heart, the parent group that gives you a list of questions, gives us the last set of questions that they think are important. How does our center compare to other centers for treating hypoplastic left heart syndrome? What are our doctor’s qualifications? All of that. So, we have their questionnaire all filled out with all the numbers from our center. So that’s in here too.”
	- Clinician 1, Family 1
*Supportive services: social work*	“And we have social workers who meet with you to make sure that we take care of all your insurance papers and they check to see if there’s anything else you need to do all of this.”
	- Clinician 1, Family 7
	“There are places to stay. Okay? There’s the Ronald McDonald House, which is pay what you can, where a lot of us can. So, people who have children in the hospital for a long time don’t have the burden of trying to find hotels and all of that. But we will have you linked with a social worker to talk about those sorts of things.”
	- Clinician 1, Family 13
*Supportive services: palliative care consultation*	“We have a group called palliative care that you can meet with. . . And they just help you process things and figure out what you want to do and what resources are available to us and things like that. So, if any time you want to meet them, we can set that up so you can meet them. Okay. Something else for you to think about, but again, you don’t have to make any decisions today.”
	- Clinician 3, Family 16
	“We have a group of people called the palliative and supportive care team. They’re, most people when they think of a palliative care team, it’s just for end-of-life stuff . . . But that’s not what they do here . . . They also support. Okay? And so, when we have big challenges, they can be really helpful in making sure we’re taking care of all the parents need . . . they see every child who gets a heart transplant.”
	- Clinician 1, Family 20

## Key Message

This study is a secondary analysis of qualitative data derived from initial fetal cardiology consultations. It describes, for the first time, how fetal cardiology clinicians discuss and frame various management options, anticipated outcomes, and supportive services.
